# Cabozantinib for metastatic breast carcinoma: results of a phase II placebo-controlled randomized discontinuation study

**DOI:** 10.1007/s10549-016-4001-y

**Published:** 2016-10-06

**Authors:** Sara M. Tolaney, Hovav Nechushtan, Ilan-Gil Ron, Patrick Schöffski, Ahmad Awada, Chris A. Yasenchak, A. Douglas Laird, Bridget O’Keeffe, Geoffrey I. Shapiro, Eric P. Winer

**Affiliations:** 1 Department of Medical Oncology, Breast Oncology Center, Dana-Farber Cancer Institute, 450 Brookline Avenue, Boston, MA 02215 USA; 2 Department of Medicine, Brigham and Women’s Hospital, Boston, MA USA; 3 Oncology Department, Hadassah Hebrew University Medical Center, Jerusalem, Israel; 4 Department of Oncology, Tel Aviv Sourasky Medical Center, Sackler Faculty of Medicine, Tel Aviv University, Tel Aviv, Israel; 5 Department of General Medical Oncology, Leuven Cancer Institute, KU Leuven, Louvain, Belgium; 6 Medical Oncology, Institut Jules Bordet, Universite Libre de Bruxelles, Brussels, Belgium; 7 Willamette Valley Cancer Institute, RiverBend Pavilion, Springfield, OR USA; 8 Exelixis, South San Francisco, CA USA

**Keywords:** Metastatic breast cancer, Cabozantinib, Vascular endothelial growth factor receptor, Progression-free survival, Overall survival, Tumor response

## Abstract

**Purpose:**

Cabozantinib (XL184), a multi-targeted oral tyrosine kinase inhibitor with activity against MET, VEGFR2, AXL, and other tyrosine kinases, was assessed in a cohort of metastatic breast cancer (MBC) patients in a phase II randomized discontinuation trial (RDT).

**Methods:**

Patients received 100 mg cabozantinib daily during a 12-week lead-in stage. Those with stable disease per modified Response Evaluation Criteria in Solid Tumors version 1.0 at 12 weeks were randomized to either continue cabozantinib or receive placebo. Primary endpoints were objective response rate (ORR) during the 12-week lead-in stage and progression-free survival (PFS) after randomization. Patients were also followed for overall survival (OS).

**Results:**

Forty-five patients with MBC and a median of three prior lines of chemotherapy for metastatic disease were enrolled. The ORR during the lead-in stage was 13.6 % (95 % confidence interval [CI] 6–25.7 %), and the disease control rate at week 12 was 46.7 % (95 % CI 31.7–61.6 %). Per the initial RDT study design, patients with stable disease at week 12 were randomized to cabozantinib or placebo. Following a Study Oversight Committee recommendation, randomization was suspended. Patients in the lead-in stage continued on open-label cabozantinib. Patients in the randomization stage were subsequently unblinded. The overall median PFS for all MBC patients was 4.3 months. Median OS was 11.4 months (95 % CI 10.5–16.5 months). The most common grade 3/4 adverse events in the lead-in stage were palmar-plantar erythrodysesthesia (13 %) and fatigue (11 %). One death from respiratory failure was reported as drug-related during the lead-in stage.

**Conclusions:**

In heavily pretreated MBC patients, cabozantinib monotherapy demonstrated clinical activity including objective response and disease control.

## Introduction

Cabozantinib is an orally bioavailable tyrosine kinase inhibitor with potent activity against MET, vascular endothelial growth factor receptor-2 (VEGFR2), AXL, and other receptor tyrosine kinases (RTKs) that have also been implicated in tumor pathobiology, including RET, KIT, and FLT3 [1]. In vivo, cabozantinib suppresses MET and VEGFR2 signaling, rapidly inducing endothelial and tumor cell apoptosis, resulting in tumor regression in various xenograft models [1].

MET and its ligand hepatocyte growth factor (HGF) have been implicated in diverse aspects of tumor pathobiology, including tumor growth, survival, angiogenesis, invasion, and dissemination [2]. Molecular alterations directly affecting MET are relatively rare in breast cancer (BC). For example, molecular inversion probe arrays of 971 early BCs revealed that 8 % exhibited an elevation of c-Met copy number [3]. However, MET pathway activation and dysregulation are implicated via other mechanisms in BC pathobiology. MET and HGF expression is significantly higher in BC cells than in adjacent non-tumor cells in surgical specimens [4]. High tumor MET expression was evident in 89 of 170 (52 %) patients with triple-negative BC and predicted shorter survival [5]. In tumor tissue from node-positive BC patients, MET overexpression was associated with poor clinical outcome independent of human epidermal growth factor receptor-2 (HER2) status [6]. Elevated levels of circulating HGF are also common and are an independent prognostic indicator associated with worse outcome in primary BC patients [7].

The VEGF receptors and ligands are central mediators of tumor neoangiogenesis and lymphangiogenesis [8]. High tumor microvessel density is predictive of poor relapse-free and overall survival (OS) in BC [9]. Moreover, elevated VEGF expression is associated with decreased relapse-free and OS in early stage BC [10, 11]. Bevacizumab, an anti-VEGF monoclonal antibody, was initially authorized by the United States Food and Drug Administration (FDA) for use in combination with chemotherapy as first-line therapy for metastatic breast cancer (MBC) based on significantly improved progression-free survival (PFS) demonstrated in several studies [12, 13]. However, the substantial PFS benefit with bevacizumab did not translate into prolonged OS, and the BC indication was later withdrawn in the United States. It is now evident that inhibition of VEGF signaling alone, although initially effective in slowing tumor growth, often culminates in emergence of an evasive resistance phenotype that ultimately promotes tumor invasiveness and metastasis [14]. MET has been implicated in such resistance in multiple preclinical models, and, in a pancreatic neuroendocrine tumor model, combined inhibition of VEGFR and MET results in enhanced efficacy over inhibition of either pathway alone [15–18].

Based on the broad preclinical and clinical activities of cabozantinib, a phase II randomized discontinuation trial (RDT) was conducted in nine selected tumor types: MBC, castration-resistant prostate cancer, hepatocellular carcinoma, non-small cell lung cancer, ovarian cancer, melanoma, pancreatic cancer, small cell lung cancer, and gastric/gastroesophageal junction cancer (ClinicalTrials.gov NCT00940225) [19, 20]. We herein describe the results for the cohort of patients with MBC.

## Patients and methods

### Patients

Eligible patients had MBC that was estrogen receptor-positive (ER^+^), ER/progesterone receptor (PgR)/HER2-negative (triple-negative), or inflammatory (regardless of receptor status) with measurable disease by modified Response Evaluation Criteria in Solid Tumors version 1.0 (mRECIST) and evidence of progression on computed tomography (CT), magnetic resonance imaging (MRI), or bone scan at screening. Additional inclusion criteria were Eastern Cooperative Oncology Group performance status (ECOG PS) 0 or 1, adequate hematologic and end-organ function, and no more than four prior standard chemotherapy regimens completed ≥4 weeks before study entry. Patients must have discontinued fulvestrant ≥4 weeks prior and other endocrine therapy ≥2 weeks prior to first dose of study drug. Patients with active brain metastases, radiation therapy within 2 weeks, or clinically significant intercurrent illness were excluded. The study was conducted in accordance with the Declaration of Helsinki and Good Clinical Practice guidelines. The study protocol and informed consent documents were reviewed and approved by the participating institutions’ Institutional Review Boards, and all patients provided consent before any study-specified procedures.

### Study design

**Fig. 1:**
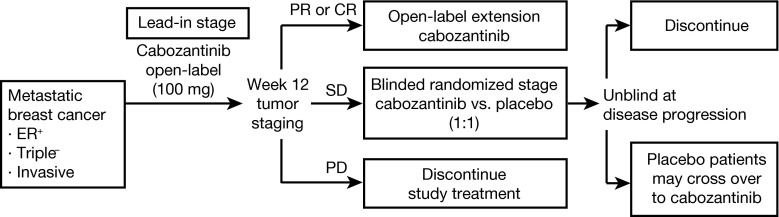
Schematic of randomized discontinuation trial (RDT) design. *ER* estrogen receptor, *CR* complete response, *PD* progressive disease, *PR* partial response, *SD* stable disease

The primary objective was to evaluate the antitumor activity of cabozantinib in multiple solid tumors including MBC. The primary endpoints were objective response rate (ORR) during the 12-week lead-in stage and progression-free survival (PFS) post-randomization. Secondary objectives assessed the safety and tolerability of the agent and potential pharmacodynamic effects. The study was designed as a randomized discontinuation trial (RDT) (Fig. 1). Patients received cabozantinib at a daily oral dose of 100 mg (freebase weight) during a 12-week, open-label, lead-in stage. Cabozantinib dosing was delayed or the daily dose reduced if clinically significant toxicities developed. Dosing interruption for ≤6 weeks was allowed.

Laboratory tests for safety monitoring were performed every 2 weeks through week 12, then every 3 weeks. Tumors were assessed every 6 weeks throughout the study. At week 12, patients with evidence of response by mRECIST remained on open-label cabozantinib, and patients with progression discontinued. Patients with stable disease at week 12 were randomized to either continued cabozantinib or placebo in double-blinded fashion. All randomized patients were followed until progression, at which point treatment assignment was unblinded, patients receiving cabozantinib were discontinued, and patients receiving placebo were allowed to restart cabozantinib and were then followed until subsequent progression. The protocol was amended to add follow-up for OS.

### Study assessments

Efficacy assessments included radiographic soft tissue and bone imaging. After enrollment of the first 20 patients, a protocol amendment required bone scans at baseline and follow-up assessments for patients with known bone metastases. The PFS analysis was conducted based on investigator-assessed response by mRECIST.

Other clinical assessments included medical and cancer history, physical examination, vital signs and body weight, electrocardiography, ECOG PS, safety laboratory values (serum chemistry, hematology, coagulation, urinalysis), concomitant medications, adverse events (AEs), and information on subsequent anticancer treatment.

### Study oversight

A Study Oversight Committee (SOC) monitored efficacy during the lead-in stage. An independent data monitoring committee monitored safety during the blinded randomized stage.

### Statistical considerations

The adaptive design study assumed that a stable disease rate of 35 % in a cohort at week 12 would be of interest. Up to 200 patients could be enrolled to a tumor-type cohort to randomize 70 patients and achieve 52 events post-randomization. This design had an 80 % power to detect a hazard ratio of 0.5 for PFS post-randomization. Cohort enrollment could be halted by the SOC if an insufficient number of patients had disease stabilization because of either high or low rates of clinical activity during the lead-in stage. The SOC generally evaluated efficacy in increments of 20 patients, but their evaluations were not based on patient completion of week 12.

The Kaplan–Meier method estimated medians for the analysis of PFS from date of randomization and OS from date of first dose. The estimation method described by Ratain et al. [21] was used for the analysis of overall PFS from the date of first dose, including the lead-in stage. All treated patients contributed to the PFS estimate through 12 weeks; thereafter, the PFS was estimated as a weighted average of those continuing on open-label treatment and those randomized to cabozantinib. The weights corresponded to the fraction of patients continuing on open-label treatment at week 12 and the proportion of patients randomized at week 12 (including patients randomized to placebo).

## Results

### Patients and treatment

**Fig. 2:**
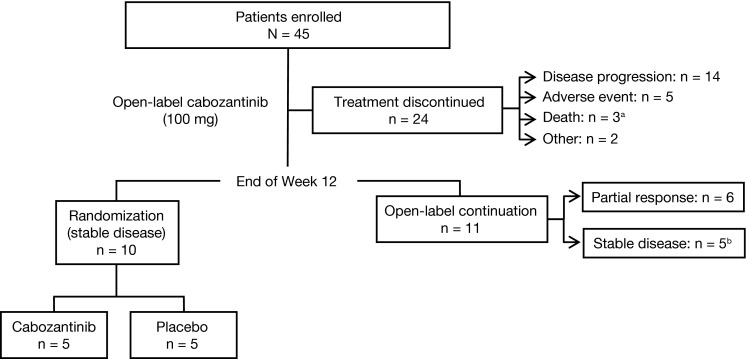
CONSORT diagram: disposition of enrolled metastatic breast cancer patients. ^a^Two deaths were disease progression, and the third death was reported as respiratory compromise. ^b^Patients with stable disease at week 12 continued on open-label cabozantinib after randomization was suspended upon recommendation of the independent study oversight committee

From November 2009 until October 2011, 45 patients with MBC were enrolled in the United States, Belgium, and Israel. Median follow-up was 14 months. Table 1 summarizes baseline demographic and clinical characteristics. Forty-three patients (96 %) were ER^+^, and two patients were triple-negative. Lung, liver, and bone metastases were present at baseline in 18 (40 %), 28 (62.2 %), and 33 (73 %) patients, respectively. Patients were heavily pretreated: 93 % with hormonal therapy, 53 % with three or more lines of chemotherapy, 80 % with taxanes, 71 % with anthracyclines, and 42 % with bevacizumab (Table 1). The SOC recommended suspension of enrollment and randomization of the MBC cohort after clinically significant efficacy was observed in multiple tumor-type cohorts (in the overall RDT), including MBC, due to the high disease control rate (DCR) and rapid symptomatic progression of many patients after randomization to placebo at week 12. Before this decision, 10 patients with stable disease at week 12 were randomized to either cabozantinib or placebo. Eleven patients continued open-label cabozantinib after week 12 (Fig. 2). Twenty-four (53 %) patients discontinued study treatment during the lead-in stage (≤12 weeks); five of these discontinued because of an AE.

**Table 1:** Baseline demographic and clinical characteristics of patients (*N* = 45)

	*n*	%
Age, years		
Median	56	
Range	27–74	
Receptor status		
ER^+^/HER2^−^	35	78
ER^+^/HER2^+^	7	16
ER^+^/HER2 unknown	1	2
Triple-negative	2	4
Histologic subtype		
Invasive ductal	37	82
Invasive lobular	3	7
Other	5	11
Visceral metastases		
Lung metastases	18	40
Liver metastases	28	62
Bone metastases	33	73
Prior hormonal therapy	42	93
Prior lines of chemotherapy		
0	1	2
1	4	9
2	16	36
≥3	24	53
Prior taxane therapy	36	80
Prior anthracycline therapy	32	71
Prior bevacizumab	19	42

*ER* estrogen receptor, *HER2* human epidermal growth factor receptor-2, *VEGF* vascular endothelial growth factor

### Tumor response in the 12-week lead-in stage

**Fig. 3:**
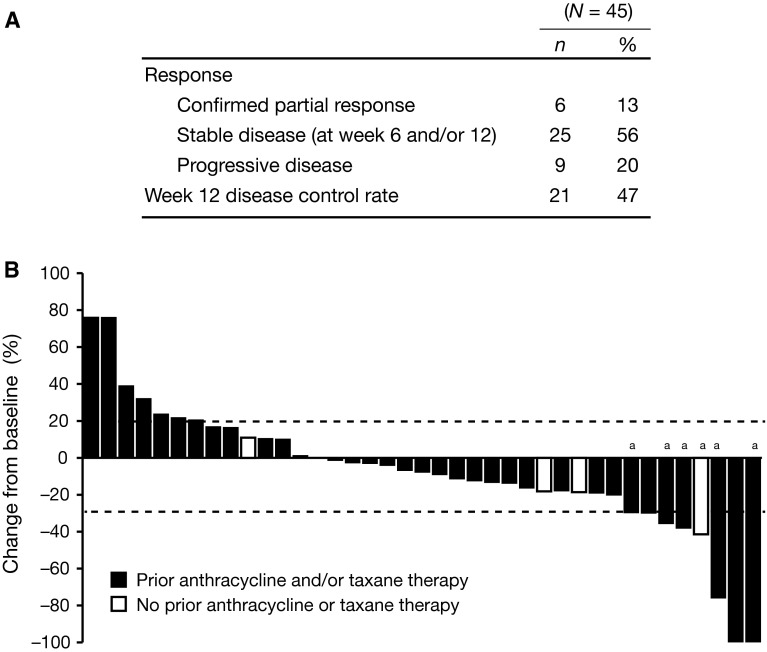
**a** Summary of modified RECIST version 1.0 (mRECIST) response. All responses were confirmed by week 18. Disease control rate is the sum of patients with confirmed partial response or stable disease at week 12. **b** Best change from baseline in investigator-assessed measurements of soft-tissue lesions using mRECIST was determined for patients who had baseline and at least one post-baseline radiographic scan in the first 12 weeks (*n* = 39). ^a^Confirmed partial response. RECIST, Response Evaluation Criteria in Solid Tumors

The ORR per mRECIST during the 12-week lead-in stage was 13.6 % (95 % CI 6–25.7 %); six patients achieved confirmed partial response. The DCR, defined as the incidence of partial response or stable disease at week 12, was 46.7 % (95 % CI 31.7–61.6 %). Twenty-five patients had stable disease and nine patients had progressive disease as their best overall response. Five patients were not evaluable for response because baseline or post-baseline assessments were unavailable. Reduction of target lesions as a best overall response was documented for 64 % (25/39) of evaluable patients (Fig. 3).

### Progression-free and overall survival

**Fig. 4:**
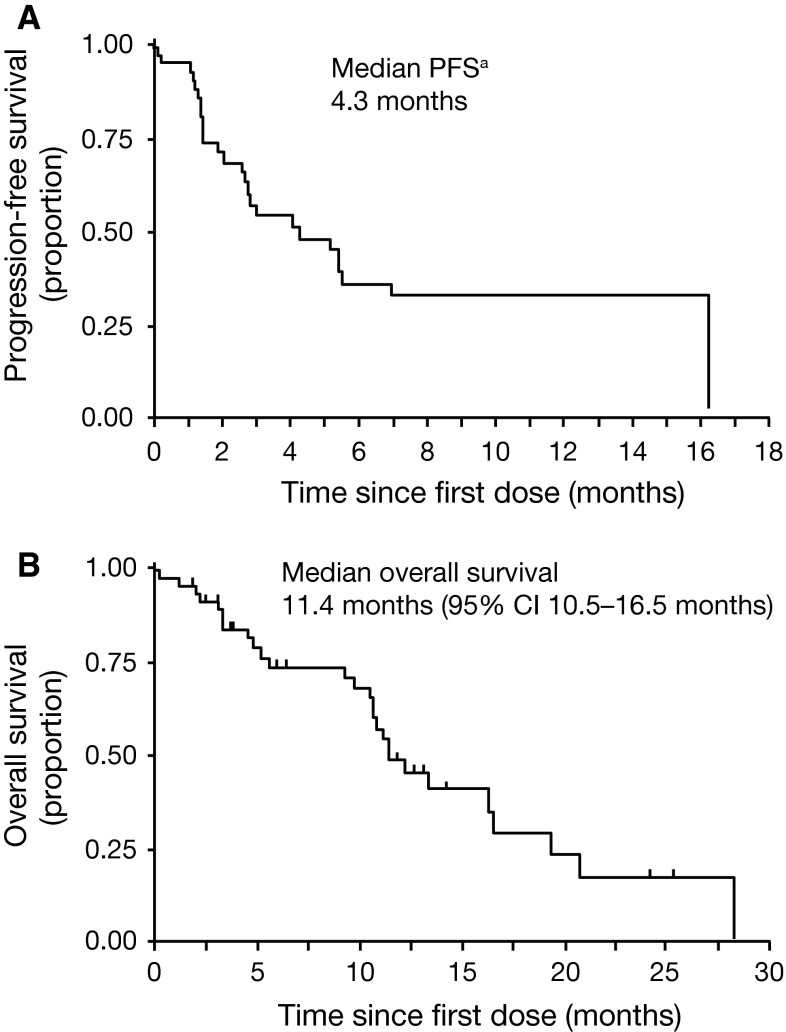
**a** Estimated overall progression-free survival for 45 metastatic breast cancer patients, determined as described by Ratain et al. [21]. **b** Overall survival (26 death events and 19 patients censored). ^a^See “Patients and Methods” section for details on the PFS calculations

Before suspension of randomization in the MBC cohort, 10 patients who had stable disease at week 12 were randomized (five cabozantinib, five placebo). Due to the small sample size, the estimated PFS post-randomization is not reported. The estimated median overall PFS for all patients from study initiation was 4.3 months (Fig. 4a) [21]. Median OS was 11.4 months (Fig. 4b) [21].

### Safety

Table 2 summarizes AEs reported during the lead-in stage of the study regardless of attribution. All patients had at least one AE, and most experienced more than one event. The most common grade ≥3 events were palmar-plantar erythrodysesthesia (PPE), fatigue, hypertension, abdominal pain, asthenia, dyspnea, and increased aspartate aminotransferase. One related grade 5 event of respiratory failure was reported in a patient who progressed with multiple pleural effusions that tested positive for malignant cells and small pulmonary nodules suspicious for metastatic disease. During the 12-week lead-in stage, five patients (11 %) discontinued treatment because of an AE (Fig. 3), and 18 (40 %) had at least one dose reduction for any reason other than patient error or noncompliance.

**Table 2:** Most frequently reported adverse events during lead-in stage regardless of causality (*N* = 45)

Adverse event^b^	All grades		Grade ≥3^a^	
	*n*	%	*n*	%
Fatigue	32	71	5	11
Nausea	29	64	1	2
Diarrhea	25	56	2	4
PPE	20	44	6	13
Decreased appetite	20	44	1	2
Vomiting	17	38	1	2
Constipation	13	29	2	4
Hypertension	11	24	3	7
Decreased weight	11	24	2	4
Dysgeusia	11	24	–	–
Dysphonia	11	24	–	–
Abdominal pain	10	22	3	7
Asthenia	10	22	3	7
Headache	10	22	–	–
Dyspnea	9	20	3	7
Dizziness	8	18	–	–
Hypothyroidism	8	18	–	–
Mucosal inflammation	8	18	–	–
Back pain	7	16	2	4
Leukopenia	7	16	1	2
Stomatitis	7	16	1	2
Pain in extremities	7	16	–	–
Pain	6	13	2	4
Alopecia	6	13	–	–
Dyspepsia	6	13	–	–
Rash	6	13	–	–
Urinary tract infection	6	13	–	–
Increased AST	5	11	3	7
Thrombocytopenia	5	11	1	2
Dry mouth	5	11	–	–
Hair color changes	5	11	–	–
Neutropenia	5	11	–	–
Oral pain	5	11	–	–

*AST* aspartate aminotransferase, *PPE* palmar-plantar erythrodysesthesia

^a^One related grade 5 event was reported: respiratory compromise

^b^MedDRA v. 14.1 Preferred Terms (converted to US spelling), CTCAE v. 3.0 grading

## Discussion

In this phase II RDT, cabozantinib demonstrated clinical activity in a heavily pretreated cohort of 45 MBC patients, with an ORR of 13.6 % and week 12 DCR of 46.7 %. Most patients had ER^+^ disease (96 %) and a median of three prior lines of chemotherapy. Of the six patients with confirmed responses, five had ER^+^, HER2^−^ breast cancer, and one patient had ER^+^, HER2^+^ disease.

Multiple RTKIs are in development for the treatment of BC, but many of them have failed to demonstrate single-agent efficacy. The success of these RTKIs may have been hindered by the dependence of the malignancy on multiple molecular pathways and by activation of compensatory mechanisms. Antiangiogenic therapy for BC has gone through a reversal with the FDA withdrawal of approval for bevacizumab in this indication. However, combining anti-VEGF pathway therapy with MET inhibition may help overcome some of the resistance and escape mechanisms that develop during treatment with anti-VEGF pathway therapy alone. In particular, inhibition of MET may suppress the induction of MET-dependent tumor invasiveness associated with VEGF blockade [14, 22]. Therefore, the ability of cabozantinib to target multiple RTKs implicated in cancer pathobiology, including MET and VEGFR2, represents a potential advantage over agents directed against VEGFR2 alone.

Clinical trials have examined the efficacy of a MET inhibitor in combination with antiangiogenic therapy in patients with triple-negative BC and, interestingly, the addition of onartuzumab to either paclitaxel and bevacizumab or paclitaxel alone did not improve PFS or OS compared with paclitaxel and bevacizumab; furthermore, the efficacy in the MET-positive and MET-negative subgroups was similar [23]. Additionally, a study that examined the MET tyrosine kinase inhibitor tivantinib in patients with metastatic triple-negative BC failed to demonstrate significant activity as monotherapy [24]. However, both of these studies of MET inhibition were conducted in patients with triple-negative disease; in contrast, our study predominantly enrolled patients with HR^+^ BC.

In summary, the clinical activity of cabozantinib observed in this first study of patients with heavily pretreated MBC is encouraging and supports further testing of cabozantinib in BC. Cabozantinib is currently FDA approved for metastatic medullary thyroid cancer and renal cell carcinoma, and studies are ongoing to further assess this agent in MBC. These studies include an assessment of cabozantinib alone and in combination with fulvestrant in patients with metastatic HR^+^ disease and known bone metastases (ClinicalTrials.gov NCT01441947), a study of cabozantinib monotherapy in patients with triple-negative MBC (ClinicalTrials.gov NCT01738438), and a study of cabozantinib (with trastuzumab for HER2^+^ disease) in BC patients with brain metastasis (ClinicalTrials.gov NCT02260531).

## Abbreviations

AE: Adverse event
BC: Breast cancer
CI: Confidence interval
CT: Computed tomography
DCR: Disease control rate
ECOG PS: Eastern Cooperative Oncology Group performance status
ER^+^: Estrogen receptor-positive
FDA: United States Food and Drug Administration
HGF: Hepatocyte growth factor
HER2: Human epidermal growth factor receptor-2
MBC: Metastatic breast cancer
mRECIST: Modified Response Evaluation Criteria in Solid Tumors
MRI: Magnetic resonance imaging
ORR: Objective response rate
OS: Overall survival
PFS: Progression-free survival
PgR: Progesterone receptor
PPE: Palmar-plantar erythrodysesthesia
RDT: Randomized discontinuation trial
RTK: Receptor tyrosine kinases
SOC: Study Oversight Committee
VEGFR2: Vascular endothelial growth factor receptor-2
